# Systemic Inflammatory Biomarkers and Chest CT Findings as Predictors of Acute Limb Ischemia Risk, Intensive Care Unit Admission, and Mortality in COVID-19 Patients

**DOI:** 10.3390/diagnostics12102379

**Published:** 2022-09-30

**Authors:** Emil Marian Arbănași, Ioana Halmaciu, Réka Kaller, Adrian Vasile Mureșan, Eliza Mihaela Arbănași, Bogdan Andrei Suciu, Cătălin Mircea Coșarcă, Ioana Iulia Cojocaru, Razvan Marian Melinte, Eliza Russu

**Affiliations:** 1 Clinic of Vascular Surgery, Mures County Emergency Hospital, 540136 Targu Mures, Romania; 2 Department of Anatomy, George Emil Palade University of Medicine, Pharmacy, Science, and Technology of Targu Mures, 540139 Targu Mures, Romania; 3 Department of Radiology, Mures County Emergency Hospital, 540136 Targu Mures, Romania; 4 Department of Surgery, George Emil Palade University of Medicine, Pharmacy, Science, and Technology of Targu Mures, 540139 Targu Mures, Romania; 5 Faculty of Pharmacy, George Emil Palade University of Medicine, Pharmacy, Science, and Technology of Targu Mures, 540139 Targu Mures, Romania; 6 First Clinic of Surgery, Mures County Emergency Hospital, 540136 Targu Mures, Romania; 7 Department of Orthopedics, Regina Maria Health Network, 540098 Targu Mures, Romania; 8 Department of Orthopedics, Humanitas MedLife Hospital, 400664 Cluj Napoca, Romania

**Keywords:** acute limb ischemia, COVID-19, MLR, NLR, PLR, SII, SIRI, AISI

## Abstract

Background: Numerous tools, including inflammatory biomarkers and lung injury severity scores, have been evaluated as predictors of thromboembolic events and the requirement for intensive therapy in COVID-19 patients. This study aims to verify the predictive role of inflammatory biomarkers [monocyte to lymphocyte ratio (MLR), neutrophil to lymphocyte ratio (NLR), platelet to lymphocyte ratio (PLR), systemic inflammatory index (SII), Systemic Inflammation Response Index (SIRI), and Aggregate Index of Systemic Inflammation (AISI)] and the CT Severity Score in acute limb ischemia (ALI) risk, intensive unit care (ICU) admission, and mortality in COVID-19 patients.; Methods: The present study was designed as an observational, analytical, retrospective cohort study and included all patients older than 18 years of age with a diagnosis of COVID-19 infection, confirmed through real time-polymerase chain reaction (RT-PCR), and admitted to the County Emergency Clinical Hospital of Targu-Mureș, Romania, and Modular Intensive Care Unit of UMFST “George Emil Palade” of Targu Mures, Romania between January 2020 and December 2021. Results: Non-Survivors and “ALI” patients were associated with higher incidence of cardiovascular disease [atrial fibrillation (AF) *p* = 0.0006 and *p* = 0.0001; peripheral arterial disease (PAD) *p* = 0.006 and *p* < 0.0001], and higher pulmonary parenchyma involvement (*p* < 0.0001). Multivariate analysis showed a high baseline value for MLR, NLR, PLR, SII, SIRI, AISI, and the CT Severity Score independent predictor of adverse outcomes for all recruited patients (all *p* < 0.0001). Moreover, the presence of AF and PAD was an independent predictor of ALI risk and mortality. Conclusions: According to our findings, higher MLR, NLR, PLR, SII, SIRI, AISI, and CT Severity Score values at admission strongly predict ALI risk, ICU admission, and mortality. Moreover, patients with AF and PAD had highly predicted ALI risk and mortality but no ICU admission.

## 1. Introduction

The pandemic caused by SARS-CoV-2 (severe acute respiratory syndrome coronavirus 2) has affected, until the present day (26 August 2022), a total of 604,392,189 cases and caused 6,483,256 deaths [1], having a negative impact on medical activities [2,3]. Patients’ symptoms range from minor (headache, loss of taste and smell) to severe (major lung damage, admission to critical care units, the necessity of invasive mechanical ventilation, sepsis, and, more recently, thromboembolic events) [4,5,6,7,8,9,10,11,12,13].

Numerous recently published studies have demonstrated the association of severe forms of COVID-19 disease with thromboembolic events [14,15,16,17]. Moreover, critically ill patients hospitalized in intensive care units (ICUs) have up to a 30% risk of developing a thromboembolic complication [18,19,20]. The main pathological mechanisms involved in the occurrence of coagulopathy in severe cases of COVID-19 include the systemic inflammatory response and endothelial dysfunction [21,22,23].

Changes in pro-coagulant factors including fibrinogen, D-dimers, or interleukin-6 (IL-6) have been associated with a higher risk of thromboembolic events in severe COVID-19 disease [24,25,26]. Unfortunately, these pro-inflammatory markers are not routinely performed in current medical practice and frequently change when COVID-19 patients’ condition worsens, necessitating their dynamic monitoring [21,22].

Acute ischemia represents the sudden interruption of arterial flow, with an incidence of 3–14 cases per 100,000 people, and is associated with a high rate of amputation and fatality in the absence of therapeutic intervention [27,28,29,30].

Cell blood count (CBC) has recently been suggested and investigated in the case of COVID-19 patients as a diagnostic and predictive tool for detecting severe forms [31,32,33], the need for ICU admission [33,34], and the necessity for invasive mechanical ventilation (IMV) [35], as well as mortality [36,37]. Among the CBC parameters, we list the following inflammatory biomarkers: monocyte to lymphocyte ratio (MLR), neutrophil to lymphocyte ratio (NLR), platelets to lymphocyte ratio (PLR), systemic inflammatory index (SII), systemic inflammation response index (SIRI), and aggregate index of systemic inflammation (AISI), whose prognostic role has been demonstrated in the field of cardio-vascular pathology [38,39,40,41,42,43,44], kidney disease [45,46], oncology [47,48], and in the last two years, in the case of COVID-19 patients [31,32,33,34,35].

This study aims to verify the predictive role of inflammatory biomarkers (MLR, NLR, PLR, SII, SIRI, and AISI) and chest CT findings and the ALI risk, ICU admission, and mortality in COVID-19 patients.

## 2. Materials and Methods

### 2.1. Study Design

The current research was designed as an observational, analytical, retrospective cohort study and included 510 patients older than 18 years of age with a diagnosis of COVID-19 infection, confirmed through real-time-polymerase chain reaction (RT-PCR), and admitted to the County Emergency Clinical Hospital of Targu-Mureș, Romania, and Modular Intensive Care Unit of UMFST “George Emil Palade” of Targu Mures, Romania between January 2020 and December 2021.

Exclusion criteria were as follows: patients with end-stage kidney disease, active tumoral status, hematological diseases, autoimmune diseases, patients requiring ICU admission within the first 72 h, patients without a chest CT scan during the hospitalization, and patients who developed other thrombo-embolic events during hospitalization such acute myocardial infarction, stroke, or acute pulmonary embolism.

Data analysis was conducted based on the two main outcomes studied: ALI developement and mortality. For the ALI events, patients were divided into two groups named “non-ALI” and “ALI”, and for the death events, patients were divided into two groups named “Survivors” and “non-Survivors”. The ideal cut-off value for MLR, NLR, PLR, SII, SIRI, and AISI was used to calculate ALI development, ICU admission, and mortality rate.

### 2.2. Data Collection

The patients’ demographic data (age and sex) were extracted from the hospital’s electronic database. We searched for the following comorbidities in the medical history: arterial hypertension (AH), ischemic heart disease (IHD), atrial fibrillation (AF), chronic heart failure (CHF), myocardial infarction (MI), type 2 diabetes (T2D), chronic obstructive pulmonary disease (COPD), peripheral arterial disease (PAD), chronic kidney disease (CKD), cerebrovascular accident (CVA), dyslipidemia, tobacco use, obesity, and length of hospital stay. Further, we collected data from the first blood test result (hemoglobin, hematocrit, neutrophil count, lymphocyte count, monocyte count, platelet count, glucose level, cholesterol level, triglyceride level, potassium level, blood urea nitrogen level, and creatinine level).

### 2.3. Systemic Inflammatory Markers

The systemic inflammation index was determined from the first blood test result. The MLR, NLR, PLR, SII, SIRI, and AISI were calculated using the equations below:

$$\mathrm{MLR}=\frac{\mathrm{total}\;\mathrm{number}\;\mathrm{of}\;\mathrm{monocytes}}{\mathrm{total}\;\mathrm{number}\;\mathrm{of}\;\mathrm{lymphocytes}}$$

$$\mathrm{NLR}=\frac{\mathrm{total}\;\mathrm{number}\;\mathrm{of}\;\mathrm{neutrophils}}{\mathrm{total}\;\mathrm{number}\;\mathrm{of}\;\mathrm{lymphocytes}}$$

$$\mathrm{PLR}=\frac{\mathrm{total}\;\mathrm{number}\;\mathrm{of}\;\mathrm{platelets}}{\mathrm{total}\;\mathrm{number}\;\mathrm{of}\;\mathrm{lymphocytes}}$$

$$\mathrm{SII}=\frac{\mathrm{total}\;\mathrm{number}\;\mathrm{of}\;\mathrm{neutrophils}\;x\;\mathrm{total}\;\mathrm{number}\;\mathrm{of}\;\mathrm{platelets}}{\mathrm{total}\;\mathrm{number}\;\mathrm{of}\;\mathrm{lymphocytes}}$$

$$\mathrm{SIRI}=\frac{\mathrm{total}\;\mathrm{number}\;\mathrm{of}\;\mathrm{neutrophils}\;x\;\mathrm{total}\;\mathrm{number}\;\mathrm{of}\;\mathrm{monocytes}}{\mathrm{total}\;\mathrm{number}\;\mathrm{of}\;\mathrm{lymphocytes}}$$

$$\mathrm{AISI}=\frac{\mathrm{total}\;\mathrm{number}\;\mathrm{of}\;\mathrm{neutrophils}\;x\;\mathrm{total}\;\mathrm{number}\;\mathrm{of}\;\mathrm{platelets}\;x\;\mathrm{total}\;\mathrm{number}\;\mathrm{of}\;\mathrm{monocytes}}{\mathrm{total}\;\mathrm{number}\;\mathrm{of}\;\mathrm{lymphocytes}}$$

### 2.4. Acute Limb Ischemia Diagnosis

Acute limb ischemia was initially diagnosed clinically, in the absence of a palpable pulse, and by using duplex ultrasound. Furthermore, in the absence of arterial flow on DUS imaging, a Computer Tomography Angiography was performed, which gave information on the arterial segment involved. The Rutherford Classification was used to determine the severity of ALI [49], and for arterial occlusion level, the arterial axis of the lower limb was divided into four segments: aorto-iliac, femoral (common femoral artery, deep femoral artery, and superficial femoral artery), popliteal, and infrapopliteal (all below the knee arteries).

### 2.5. Chest CT Findings

Chest CT exams were performed to quantify the extent of pulmonary parenchymal involvement based on visual assessment for each lobe. Image analysis was performed using a PACS (Picture Archiving and Communication System) workstation (INFINITT Healthcare Co., Ltd., Seoul, Korea). Furthermore, the presence of ground-glass opacities, consolidation, pleural effusion, and crazy paving, was quantified.

The severity of lung lesions was calculated by quantifying the disease-affected areas for each lobe to evaluate pulmonary parenchymal involvement. Each of the five lobes was given a score ranging from 0 to 5, based on the percentage of the affected area as follows: none (0%), score 1 (<5% involvement), score 2 (5–25% involvement), score 3 (26–49% involvement), score 4 (50–75% involvement), and score 5 (>75% involvement). The severity of lung lesions was calculated by adding the values for five lobes ranging from 0 to 25.

### 2.6. Study Outcomes

The primary endpoints were ALI development, intensive care unit admission, and in-hospital mortality rate. Outcomes were stratified for the optimal MLR, NLR, PLR, SII, SIRI, AISI, and CT Severity Score cut-off value at baseline. The secondary endpoints were the ICU admission and in-hospital mortality rate for ALI patients.

### 2.7. Statistical Analysis

SPSS for Mac OS version 28.0.1.0 was used for the statistical analysis (SPSS, Inc., Chicago, IL, USA), and Chi-square tests were employed to analyze the associations of MLR, NLR, PLR, SII, SIRI, and AISI with the category factors, while Student’s t and Mann–Whitney tests were used to assess differences in continuous variables. The receiver operating characteristic (ROC) was used to assess the predictive power and to establish the cut-off values of MLR, NLR, PLR, SII, SIRI, and AISI, based on the Youden index (Youden Index = Sensitivity + Specificity − 1, ranging from 0 to 1). To identify independent predictors of ALI development, ICU admission, and mortality, a multivariate logistic regression analysis using variables with *p* < 0.1 was undertaken.

## 3. Results

### 3.1. Baseline Characteristics of All Patients, Classified According to the ALI Risk

During the study period, 510 patients diagnosed with COVID-19 met the inclusion criteria and were followed up during hospitalization. The mean age was 70.44 ± 11.05 (25–94), and 305 patients were male (59.80%). During the hospitalization, 49 (9.61%) patients developed ALI, 187 (36.67%) needed ICU, and 114 (22.35%) died. The rest of the comorbidities, chest CT findings, and laboratory data are presented in Table 1.

Regarding the ALI risk, the male sex had a lower incidence in the ALI group (*p* = 0.01), a higher incidence of AF (*p* = 0.0006), and PAD (*p* = 0.006). Furthermore, each pulmonary lobe had a higher incidence of parenchymal involvement (*p* < 0.0001), as well as a higher CT Severity Score (*p* < 0.0001) in the second group. In terms of laboratory findings, sever variables were associated with ALI development: ALI patients had lower hemoglobin (*p* = 0.03), hematocrit (*p* = 0.03), cholesterol (*p* = 0.01), triglyceride (*p* = 0.01), and lymphocyte (*p* < 0.0001) levels and higher neutrophils (*p* < 0.0001), PLT (*p* < 0.0001), BUN (*p* = 0.04), and glucose (*p* = 0.0004). All of the systemic inflammatory markers and outcomes were higher in the ALI (*p* < 0.0001) group, as seen in Table 1.

### 3.2. Baseline Characteristics of All Patients, Classified by Mortality Risk

Depending on the survival status during the hospitalization, the mean age was statistically higher in the second group (*p* = 0.001). However, the male sex had a lower incidence in the second group (*p* = 0.02). In terms of comorbidities, in the non-Survivors’ group, there was a higher incidence of AF (*p* = 0.0001) and PAD (*p* < 0.0001). Regarding the chest CT findings, the non-survivors had higher parenchymal pulmonary involvement (*p* < 0.0001), incidence of consolidation (*p* = 0.03), pleural effusion (*p* = 0.03), GGO (*p* = 0.0001), and crazy paving (*p* = 0.0005). Moreover, several variables from laboratory data were associated with poor outcomes: non-Survivors had lower hemoglobin (*p* < 0.0001), hematocrit (*p* < 0.0001), and lymphocyte (*p* < 0.0001) levels and higher neutrophil (*p* < 0.0001), monocyte (*p* = 0.0002), PLT (*p* = 0.0006), glucose (*p* < 0.0001), and BUN (*p* = 0.01) levels (Table 2). All of the systemic inflammatory markers and outcomes were higher in the non-Survivors’ (*p* < 0.0001) group, as seen in Table 2.

### 3.3. ROC Curves, Optimal Cut-Off Values, AUC, Predictive Accuracy of Inflammatory Markers, and CT Severity Score

**Figure 1:**
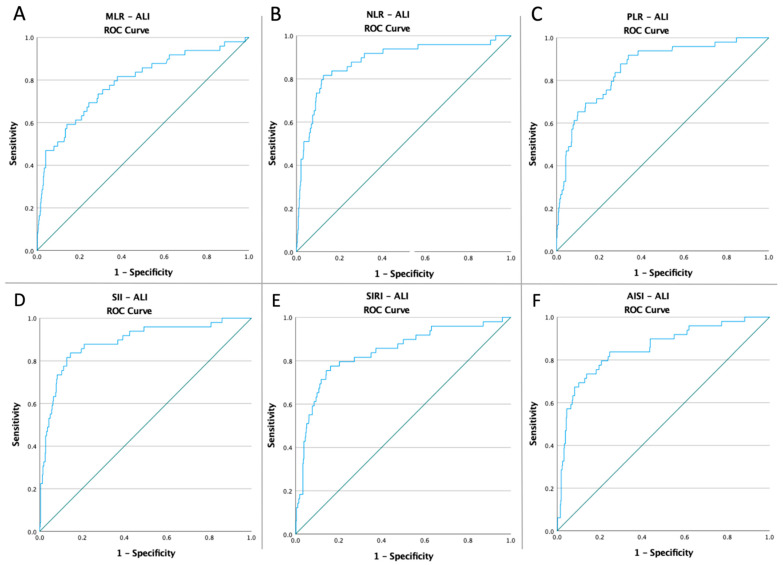
ROC curve analysis concerning the ALI risk (**A**) MLR (AUC: 0.829; *p* < 0.0001), (**B**) NLR (AUC: 0.856; *p* < 0.0001), (**C**) SII (AUC: 0.858; *p* < 0.0001), (**D**) SIRI (AUC: 0.785; *p* < 0.0001), (**E**) AISI (AUC: 0.765; *p* < 0.0001), and (**F**) TSS (AUC: 0.759; *p* < 0.0001).

**Figure 2:**
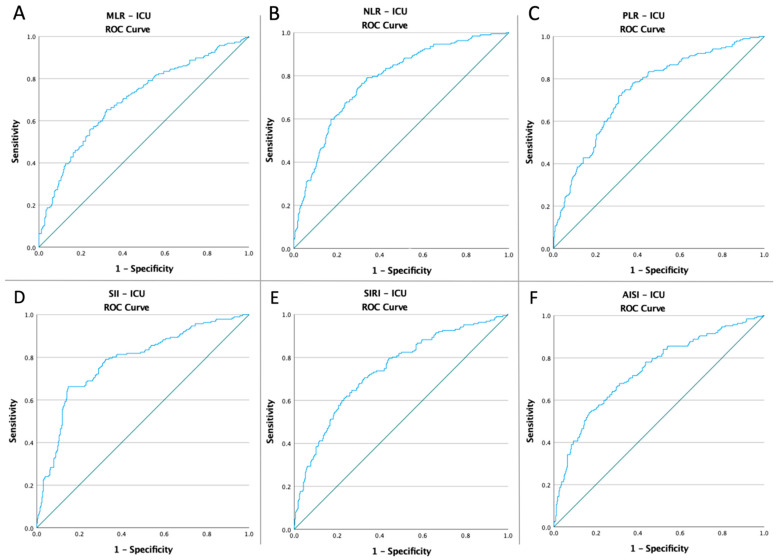
ROC curve analysis concerning ICU admission (**A**) MLR (AUC: 0.829; *p* < 0.0001), (**B**) NLR (AUC: 0.856; *p* < 0.0001), (**C**) SII (AUC: 0.858; *p* < 0.0001), (**D**) SIRI (AUC: 0.785; *p* < 0.0001), (**E**) AISI (AUC: 0.765; *p* < 0.0001), and (**F**) TSS (AUC: 0.759; *p* < 0.0001).

**Figure 3:**
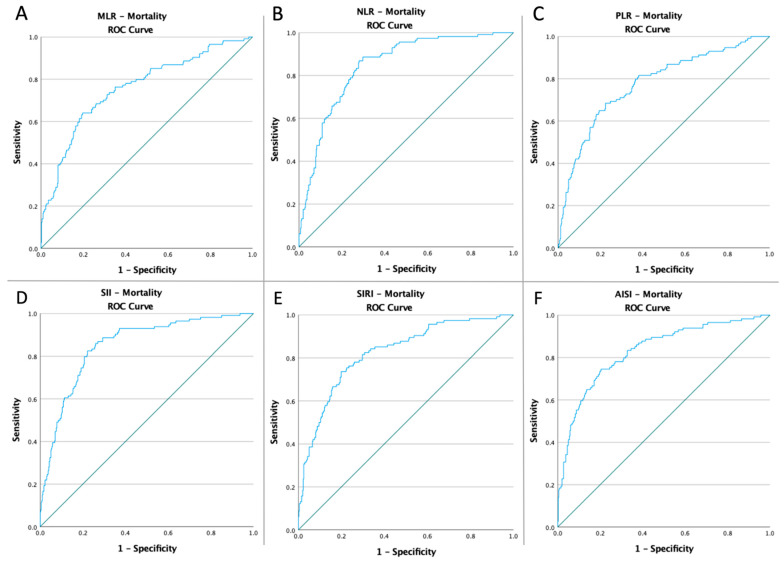
ROC curve analysis concerning the mortality (**A**) MLR (AUC: 0.829; *p* < 0.0001), (**B**) NLR (AUC: 0.856; *p* < 0.0001), (**C**) SII (AUC: 0.858; *p* < 0.0001), (**D**) SIRI (AUC: 0.785; *p* < 0.0001), (**E**) AISI (AUC: 0.765; *p* < 0.0001), and (**F**) TSS (AUC: 0.759; *p* < 0.0001).

**Figure 4:**
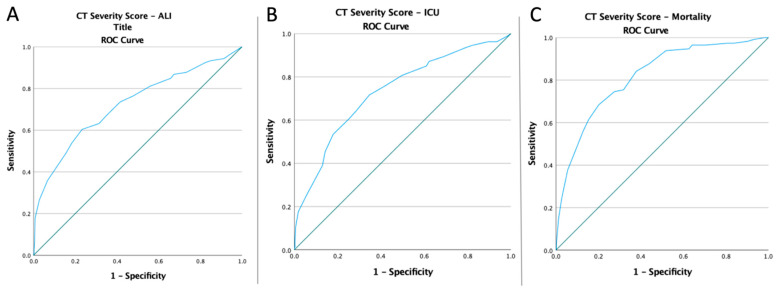
ROC curve analysis for the CT Severity Score (**A**) concerning the ALI risk (AUC: 0.725; *p* < 0.0001), (**B**) concerning the ICU admission (AUC: 0.733; *p* < 0.0001), and (**C**) concerning the mortality rate (AUC: 0.816; *p* < 0.0001).

Receiver operating characteristic curves of inflammatory biomarkers and the CT global score were created to determine whether the baseline of these markers was predictive of ALI risk, ICU admission, and mortality in COVID-19 patients (Figure 1, Figure 2, Figure 3 and Figure 4). The optimal cut-off value obtained from Youden’s index, the area under the curve (AUC), and the predictive accuracy of the markers are listed in Table 3.

### 3.4. Univariate and Multivariate Analyses of Inflammatory Biomarkers, the Chest CT Severity Score, and Adverse Events in All Patients

Depending on the optimal cut-off value according to the ROC, the outcomes were further analyzed after dividing the patients into paired groups. There was a higher incidence of all adverse outcomes for all the biomarkers analyzed, as seen in Table 4.

Multivariate analysis showed that a high baseline value for all the analyzed markers was an independent predictor of ALI risk: MLR (OR:4.37; *p* < 0.001), NLR (OR:8.55; *p* < 0.001), PLR (OR:12.95; *p* < 0.001), SII (OR:15.70; *p* < 0.001), SIRI (OR:6.81; *p* < 0.001), AISI (OR:7.46; *p* < 0.001), and the CT Severity Score (OR:14.71; *p* < 0.001). Moreover, the high baseline value of systemic inflammatory biomarkers was an independent predictor of ICU admission (*p* < 0.001) and mortality (*p* < 0.001). Furthermore, for all hospitalized patients, AF (*p* = 0.009 and *p* < 0.0001) and PAD (*p* = 0.002 and *p* < 0.001) were independent predictors of ALI and mortality but not for ICU admission (Table 5).

### 3.5. Baseline Characteristics and Multivariate analysis of ALI Patients, Divided According to the Mortality Risk

The ALI patients were divided into two groups regarding their survival status during the hospitalization. There was a lower incidence of Rutherford class I (*p* = 0.02) and a higher incidence of Rutherford class III (*p* = 0.02) in the non-Survivors’ group. Regarding the arterial segment involved, the occlusion of infrapopliteal segments (*p* = 0.02) was higher in the non-Survivors, and the femoral segment involved (*p* = 0.003) was higher in the Survivors’ group. In multivariate analysis, the occlusion of the femoral segment acted as a protective factor against any negative adverse events during the study for ALI patients (*p* = 0.02; *p* = 0.003), as well as RC I for mortality (*p* = 0.02), but not for ICU admission (*p* = 0.054). In contrast, the RC III and the occlusion of the infrapopliteal segment were independent predictors of mortality (*p* = 0.04 and *p* = 0.02), as seen in Table 6.

## 4. Discussion

The main finding of this study is the demonstration of the predictive role of inflammatory biomarkers and the demonstration of the predictive role of the pulmonary damage score in detecting patients at risk of developing acute ischemia (all *p* < 0.001), the requirement for ICU admission (all *p* < 0.0001), and mortality (all *p* < 0.001) in the case of patients diagnosed with COVID-19. Moreover, male gender and the presence of cardiovascular comorbidities (AF and PAD) were independent prognostic factors in the case of ALI risk (*p* = 0.003; *p* < 0.001; *p* = 0.006) and mortality (*p* = 0.02; *p* < 0.001; *p* < 0.001).

Radiological tools are crucial in detecting patients with severe forms and stratifying risk groups. Saeed et al. [50] found that the CT Severity Score was strongly correlated with lymphopenia and elevated levels of inflammatory markers in 902 COVID-19 patients. Furthermore, Lieveld et al. [51] reported that the CT Severity Score can be used as an independent predictor of hospital admission (OR:1.18; *p* < 0.001), ICU admission (OR:1.23; *p* < 0.001), and 30-day mortality (OR:1.12; *p* < 0.001).

Regarding inflammatory markers, severe forms of COVID-19 infection and the unfavorable progression of the disease are associated with high levels of inflammatory markers. In the works published by Wang R et al. and Simon et al., high PLR values were associated with mortality in univariate analysis (OR:1.004; *p* < 0.001) (OR:1.001; *p* = 0.04) in the case of COVID-19 patients [52,53].

Hypercoagulability is one of the main factors involved in the etiology of severe forms of COVID-19 [54,55]. The association of SARS-CoV-2 infection with thromboembolic events is well known, with a risk of up to 30% of developing an embolic event in severe forms [14,15,16,17,18,19,20]. Moreover, Strazzulla et al. [12] discovered that the total number of neutrophils (OR:1.20; 95% CI:1.04–1.40; *p* = 0.01) and lymphocytes (OR:0.45; 95% CI:0.23–0.86; *p* = 0.01) were independently associated with acute pulmonary embolism a cohort of 184 COVID-19 patients. Furthermore, Roncati L. et al. explained the influence of an abnormal inflammatory response in severe COVID-19 patients on pro-coagulant status through platelet release [56,57,58].

Previous studies showed that the optimal cut-off values for the inflammatory biomarkers that predicted poor outcomes for non-COVID-19 patients diagnosed with ALI ranged from 4.33 to 6.67 for NLR [41,42,59,60,61] and from 143.34 to 269.9 for PLR [41,42,59,62]. The median NLR (15.16) and PLR (316.66) values of ALI patients in this study are also much higher than those in the non-COVID-19 literature.

In a study including 267 patients with COVID-19 pneumonia, Halmaciu et al. [62] found that inflammatory markers (NLR, MLR, SII, SIRI, AISI) and the lung damage score had a predictive role in the requirement of invasive mechanical ventilation (all *p* < 0.0001), and death (all *p* < 0.001). In addition to the results obtained in the previous study, the predictive role of inflammatory markers in the development of ALI was established in this paper (all *p* < 0.001), and high values of inflammatory markers had an independent predictive role in the requirement for ICU admission (all *p* < 0.001) and for mortality (all *p* < 0.001), as seen in Table 5.

To the best of our knowledge, this is the first study to evaluate the predictive relevance of hematological parameters in the development of ALI in COVID-19 patients. The importance of these hematological indicators in predicting short-term mortality, ICU admission, and the requirement of IMV has been widely researched [31,32,33,34,35,36,37,52,55].

The progression of non-critical COVID-19 patients is unpredictable, making therapy for these patients difficult for specialists. The use of diagnostic tools in risk group stratification is essential in modern medical practice, as it allows us to develop a therapeutic strategy and prevent thromboembolic events.

Our study has certain limitations, despite the statistically significant results for 510 patients. First, it is monocentric, retrospective research with a short-term follow-up. Furthermore, due to the study’s retrospective nature, we could not access data about chronic medications used before admission (such as corticosteroids and anti-inflammatory medications). Therefore, we could not establish the effect of other medications on inflammatory biomarkers. Prospective multicenter studies with long-term follow-up are recommended in the future. Furthermore, additional research is necessary to support our findings.

## 5. Conclusions

According to our findings, higher MLR, NLR, PLR, SII, SIRI, AISI, and chest, CT Severity Score values at admission strongly predict ALI risk, ICU admission, and mortality. Moreover, male sex, AF, and PAD strongly predicted ALI risk and fatality. Given the high risk of thromboembolic events and coagulopathy status in COVID-19 patients and the low cost of these ratios and chest CT pulmonary parenchymal involvement, they can be used for admission risk group categorization, improved patient care, and the development of predictive patterns.

## Figures and Tables

**Table 1:** Demographic data, comorbidities, risk factors, laboratory findings, and outcomes for all patients divided according to the ALI risk.

Variables	All Patients *n* = 510	non-ALI *n* = 461	ALI *n* = 49	*p* Value (OR; CI 95%)
Age mean ± SD (min–max)	70.44 ± 11.05 (25–94)	70 ± 11.08 (25–92)	73.89 ± 13.15 (33–94)	0.051
Male sex no. (%)	305 (59.80%)	284 (61.60%)	21 (42.85%)	0.01 (0.46; 0.25–0.84)
**Comorbidities and Risk Factors**				
AH, no. (%)	307 (60.20%)	276 (59.87%)	31 (63.27%)	0.64 (1.15; 0.62–2.12)
IHD, no. (%)	171 (33.53%)	155 (33.62%)	16 (32.65%)	0.89 (0.95; 0.51–1.79)
AF, no. (%)	132 (25.88%)	109 (23.64%)	23 (46.94%)	0.0006 (2.85; 1.56–5.20)
CHF, no. (%)	194 (38.04%)	177 (38.39%)	17 (34.69%)	0.61 (0.85; 0.45–1.58)
MI, no. (%)	147 (28.82%)	130 (28.20%)	17 (34.69%)	0.34 (1.35; 0.72–2.52)
T2D, no. (%)	196 (38.43%)	176 (38.18%)	20 (40.82%)	0.71 (1.11; 0.61–2.03)
COPD, no. (%)	94 (18.43%)	84 (18.22%)	10 (20.41%)	0.70 (1.15; 0.55–2.39)
PAD, no. (%)	217 (42.55%)	187 (40.56%)	30 (61.22%)	0.006 (2.31; 1.26–4.23)
Dyslipidemia, no. (%)	218 (42.75%)	197 (42.73%)	21 (42.86%)	0.98 (1.00; 0.55–1.82)
CKD, no. (%)	102 (20%)	90 (19.52%)	12 (24.49%)	0.40 (1.33; 0.67–2.66)
CVA, no. (%)	154 (30.20%)	134 (29.07%)	20 (40.82%)	0.09 (1.68; 0.91–3.07)
Obesity, no. (%)	142 (27.84%)	125 (27.11%)	17 (34.69%)	0.26 (1.42; 0.76–2.66)
Tobacco, no. (%)	175 (34.31%)	154 (33.41%)	21 (42.86%)	0.18 (1.49; 0.82–2.71)
**Chest CT Findings**				
Consolidation, no. (%)	148 (29.01%)	134 (29.06%)	14 (28.57%)	0.94
Pleural Effusion, no. (%)	42 (8.23%)	37 (8.02%)	5 (10.20%)	0.59
Ground Glass-Opacities, no. (%)	278 (54.5%)	246 (53.36%)	32 (65.3%)	0.11
Crazy paving, no. (%)	59 (11.56%)	51 (11.06%)	8 (16.32%)	0.27
Right Upper Lobe, median [Q1–Q3]	2 [1–3]	2 [1–3]	3 [2–4]	<0.0001
Right Middle Lobe, median [Q1–Q3]	3 [1–4]	2 [1–3]	4 [2–4]	<0.0001
Right Lower Lobe, median [Q1–Q3]	3 [2–4]	3 [2–4]	4 [3–4]	<0.0001
Left Upper Lobe, median [Q1–Q3]	2 [1–3]	2 [1–3]	3 [2–4]	<0.0001
Left Lower Lobe, median [Q1–Q3]	2 [2–4]	2 [1–3]	3 [2–4]	<0.0001
CT Severity Score, median [Q1–Q3]	12 [8–17]	11 [7–15]	17 [12–20]	<0.0001
**Laboratory Data**				
Hemoglobin g/dL, median [Q1–Q3]	13.23 [11.5–14.51]	13.35 [11.56–14.57]	12.7 [11.1–14]	0.058
Hematocrit %, median [Q1–Q3]	40.4 [35.62–44.1]	40.59 [35.9–44.11]	38.7 [31.6–43.2]	0.06
Neutrophils ×10^3^/uL, median [Q1–Q3]	6.58 [4.80–8.95]	6.26 [4.69–8.44]	11.29 [7.96–14.65]	<0.0001
Lymphocytes ×10^3^/uL, median [Q1–Q3]	1.73 [1.21–2.32]	1.78 [1.3–2.4]	0.85 [0.57–1.16]	<0.0001
Monocyte ×10^3^/uL, median [Q1–Q3]	0.63 [0.47–0.85]	0.63 [0.47–0.83]	0.70 [0.49–1.15]	0.09
PLT ×10^3^/uL, median [Q1–Q3]	243 [199–300.22]	242.9 [195.5–295.1]	278.1 [207–378]	0.007
Glucose mg/dL, median [Q1–Q3]	112 [95–142]	110 [94–138]	132.3 [103.1–169]	0.002
Cholesterol mg/dL, median [Q1–Q3]	176.05 [145.12–210]	177.3 [145.2–211.4]	160.8 [139.2–189]	0.01
Triglyceride mg/dL, median [Q1–Q3]	115.4 [90.92–159.37]	115.4 [91.4–160]	105.7 [87.3–149.2]	0.01
Potassium mmol/L, median [Q1–Q3]	4.35 [3.91–5.03]	4.35 [3.91–5.06]	4.3 [3.85–4.89]	0.25
Sodium mmol/L, median [Q1–Q3]	140 [139–142]	140 [139–142]	140 [140–142]	0.08
BUN mg/dL, median [Q1–Q3]	42.8 [32.3–55.6]	42.4 [32.2–55.1]	46.2 [34.2–72.5]	0.04
Creatinine mg/dL, median [Q1–Q3]	0.91 [0.76–1.12]	0.90 [0.75–1.11]	1 [0.8–1.24]	0.07
MLR, median [Q1–Q3]	0.35 [0.25–0.57]	0.33 [0.24–0.52]	0.81 [0.45–1.38]	<0.0001
NLR, median [Q1–Q3]	3.75 [2.28–7.06]	3.49 [2.19–6.06]	15.16 [9.40–20.26]	<0.0001
PLR, median [Q1–Q3]	138.21 [104.65–207.15]	131.96 [100.33–187.15]	316.66 [189.62–466.76]	<0.0001
SII, median [Q1–Q3]	915.68 [531.03–1781.99]	825.21 [518.37–1490.54]	3751.57 [2384.21–5769.75]	<0.0001
SIRI, median [Q1–Q3]	2.22 [1.26–5.2]	2.10 [1.20–4.20]	10.87 [6.32–13.74]	<0.0001
AISI, median [Q1–Q3]	540.05 [291.27–1340.78]	496.97 [280–1052.86]	3115.66 [1387.50–4576.21]	<0.0001
**Outcomes**				
ALI, no. (%)	49 (9.61%)	-	49 (100%)	<0.0001
ICU, no. (%)	187 (36.67%)	149 (32.32%)	38 (77.55%)	<0.0001 (7.23; 3.59–14.55)
Mortality, no. (%)	114 (22.35%)	87 (18.87%)	27 (55.10%)	<0.0001 (5.27; 2.86–9.70)
Hospital stays, day median [Q1–Q3]	8 [5–11]	8 [5–11]	8 [5–12]	0.44

AH = arterial hypertension; IHD = ischemic heart disease; AF = atrial fibrillation; CHF = chronic heart failure; MI = myocardial infarction; T2D = type 2 diabetes; COPD = chronic obstructive pulmonary disease; PAD = peripheral arterial disease; CKD = chronic kidney disease; CVA = cerebrovascular accident; PLT = total platelet count; BUN = blood urea nitrogen; MLR = monocyte to lymphocyte ratio; NLR = neutrophil to lymphocyte ratio; PLR = platelets to lymphocyte ratio; SII = systemic inflammatory index; SIRI = systemic inflammation response index; AISI = aggregate index of systemic inflammation; ALI = acute limb ischemia; ICU = intensive care unit.

**Table 2:** Demographic data, comorbidities, risk factors, laboratory findings, and outcomes for all patients according to the mortality risk.

Variables	Survivors *n* = 396	Non-Survivors *n* = 114	*p* Value (OR; CI 95%)
Age mean ± SD (min–max)	69.60 ± 10.84 (25–92)	73.35 ± 11.34 (41–94)	0.001
Male sex no. (%)	247 (62.37%)	58 (50.88%)	0.02 (0.62; 0.41–0.95)
**Comorbidities**			
AH, no. (%)	228 (57.78%)	79 (69.30%)	0.02 (1.66; 1.06–2.59)
IHD, no. (%)	138 (34.85%)	33 (28.95%)	0.24 (0.76; 0.48–1.19)
AF, no. (%)	86 (21.72%)	46 (40.35%)	0.0001 (2.43; 1.56–3.80)
CHF, no. (%)	152 (38.38%)	42 (36.84%)	0.76 (0.93; 0.60–1.44)
MI, no. (%)	116 (29.29%)	31 (27.19%)	0.66 (0.90; 0.56–1.43)
T2D, no. (%)	150 (37.88%)	46 (40.35%)	0.63 (1.10; 0.72–1.69)
COPD, no. (%)	77 (19.44%)	17 (14.91%)	0.27 (0.72; 0.40–1.28)
PAD, no. (%)	141 (35.61%)	76 (66.67%)	<0.0001 (3.61; 2.32–5.61)
Dyslipidemia, no. (%)	168 (42.42%)	50 (43.86%)	0.78 (1.06; 0.69–1.61)
CKD, no. (%)	76 (19.19%)	26 (22.81%)	0.39 (1.24; 0.75–2.05)
CVA, no. (%)	116 (29.29%)	38 (33.33%)	0.40 (1.20; 0.77–1.88)
Obesity, no. (%)	114 (28.79%)	28 (24.56%)	0.37 (0.80; 0.49–1.29)
Tobacco, no. (%)	134 (33.84%)	41 (35.96%)	0.67 (1.09; 0.71–1.69)
**Chest CT Findings**			
Consolidation, no. (%)	106 (26.76%)	42 (36.84%)	0.03
Pleural Effusion, no. (%)	27 (6.81%)	15 (13.15%)	0.03
GGO, no. (%)	197 (49.74%)	81 (71.05%)	0.0001
Crazy paving, no. (%)	35 (8.83%)	24 (21.05%)	0.0005
Right Upper Lobe, median [Q1–Q3]	2 [1–3]	3 [2–4]	<0.0001
Right Middle Lobe, median [Q1–Q3]	2 [1–3]	4 [3–4]	<0.0001
Right Lower Lobe, median [Q1–Q3]	2 [2–3]	4 [3–4]	<0.0001
Left Upper Lobe, median [Q1–Q3]	2 [1–3]	3 [2–4]	<0.0001
Left Lower Lobe, median [Q1–Q3]	2 [1–3]	4 [3–4]	<0.0001
CT Severity Score, median [Q1–Q3]	11 [7–15]	18 [14.25–19]	<0.0001
**Laboratory Data**			
Hemoglobin g/dL, median [Q1–Q3]	13.5 [11.9–14.61]	12.35 [10.1–14.07]	<0.0001
Hematocrit %, median [Q1–Q3]	40.82 [36.77–44.3]	36.84 [31.91–42.77]	<0.0001
Neutrophils ×10^3^/uL, median [Q1–Q3]	5.83 [4.52–7.77]	9.43 [7.46–13.18]	<0.0001
Lymphocytes ×10^3^/uL, median [Q1–Q3]	1.85 [1.35–2.46]	1.20 [0.82–1.70]	<0.0001
Monocyte ×10^3^/uL, median [Q1–Q3]	0.61 [0.47–0.81]	0.72 [0.52–1.12]	0.0002
PLT ×10^3^/uL, median [Q1–Q3]	238.35 [192.97–284.25]	257.5 [211.77–352]	0.0006
Glucose mg/dL, median [Q1–Q3]	106.65 [93–134]	132.65 [104.25–162.42]	<0.0001
Cholesterol mg/dL, median [Q1–Q3]	177.95 [145.97–208.4]	165.75 [142.9–214.22]	0.20
Triglyceride mg/dL, median [Q1–Q3]	117.3 [91.62–158.1]	107 [86.5–167.18]	0.24
Potassium mmol/L, median [Q1–Q3]	4.37 [3.91–4.94]	4.31 [3.85–5.13]	0.44
Sodium mmol/L, median [Q1–Q3]	140 [139–142]	141 [139–142]	0.051
BUN mg/dL, median [Q1–Q3]	41.9 [32.27–54.8]	45.5 [32.72–67.8]	0.01
Creatinine mg/dL, median [Q1–Q3]	0.9 [0.75–1.11]	0.97 [0.78–1.22]	0.06
MLR, median [Q1–Q3]	0.32 [0.23–0.47]	0.62 [0.39–0.91]	<0.0001
NLR, median [Q1–Q3]	3.01 [2.05–5.05]	8.45 [5.62–14.52]	<0.0001
PLR, median [Q1–Q3]	128.22 [94.94–168.33]	229.83 [150.97–350.71]	<0.0001
SII, median [Q1–Q3]	719.53 [482.92–1290.48]	2303.58 [1457.83–3783.06]	<0.0001
SIRI, median [Q1–Q3]	1.86 [1.11–3.42]	6.93 [3.75–12.02]	<0.0001
AISI, median [Q1–Q3]	425.93 [257.41–857.88]	2100.38 [894.26–3333.88]	<0.0001
**Outcomes**			
ALI, no. (%)	29 (7.32%)	20 (17.54%)	<0.0001 (5.74; 3.57–9.25)
ICU, no. (%)	108 (27.27%)	79 (699.3%)	<0.0001 (6.01; 3.81–9.48)
Mortality, no. (%)	-	114 (100%)	<0.0001
Hospital Stays, Day Median [Q1–Q3]	8 [5–11]	7 [4–12]	0.25

AH = arterial hypertension; IHD = ischemic heart disease; AF = atrial fibrillation; CHF = chronic heart failure; MI = myocardial infarction; T2D = type 2 diabetes; COPD = chronic obstructive pulmonary disease; PAD = peripheral arterial disease; CKD = chronic kidney disease; CVA = cerebrovascular accident; PLT = total platelet count; BUN = blood urea nitrogen; MLR = monocyte to lymphocyte ratio; NLR = neutrophil to lymphocyte ratio; PLR = platelets to lymphocyte ratio; SII = systemic inflammatory index; SIRI = systemic inflammation response index; AISI = aggregate index of systemic inflammation; ALI = acute limb ischemia; ICU = intensive care unit.

**Table 3:** ROC curves, optimal cut-off value, AUC, and predictive accuracy of inflammatory markers (MLR, NLR, PLR, SII, SIRI, and AISI) and the CT Severity Score.

Variables	Cut-Off	AUC	Std. Error	95% CI	Sensitivity	Specificity	*p* Value
	**ALI**						
**MLR** **NLR** **PLR**	0.49	0.787	0.038	0.713–0.862	71.4%	71.6%	<0.0001
	8.34	0.882	0.029	0.824–0.939	81.6%	87.4%	<0.0001
	178.99	0.858	0.028	0.803–0.912	81.6%	73.1%	<0.0001
**SII**	2219.28	0.888	0.028	0.834–0.942	81.6%	87.2%	<0.0001
**SIRI**	5.04	0.839	0.034	0.773–0.905	79.6%	79.6%	<0.0001
**AISI**	1296.62	0.851	0.032	0.789–0.913	79.6%	79.2%	<0.0001
**CT Severity Score**	15.50	0.725	0.030	0.665–0.784	60.4%	76.7%	<0.0001
	**ICU**						
**MLR** **NLR** **PLR**	0.39	0.700	0.024	0.652–0.748	65.2%	67.8%	<0.0001
	3.71	0.780	0.021	0.739–0.821	79.1%	65.9%	<0.0001
	142.61	0.743	0.022	0.699–0.787	73.8%	67.2%	<0.0001
**SII**	1413.38	0.779	0.022	0.736–0.821	66.3%	85.1%	<0.0001
**SIRI**	2.33	0.740	0.023	0.696–0.785	70.6%	67.2%	<0.0001
**AISI**	650.58	0.738	0.023	0.692–0.783	67.9%	68.7%	<0.0001
**CT Severity Score**	12.50	0.733	0.023	0.687–0.779	71.7%	65.3%	<0.0001
	**Mortality**						
**MLR** **NLR** **PLR**	0.45	0.758	0.027	0.706–0.811	68.4%	74%	<0.0001
	4.57	0.845	0.019	0.807–0.882	86.8%	72%	<0.0001
	177.51	0.775	0.026	0.724–0.825	68.4%	77.5%	<0.0001
**SII**	1346.51	0.850	0.020	0.811–0.889	82.5%	77.8%	<0.0001
**SIRI**	4.02	0.823	0.022	0.780–0.867	73.7%	80.1%	<0.0001
**AISI**	973.59	0.830	0.023	0.786–0.874	74.6%	79.5%	<0.0001
**CT Severity Score**	14.50	0.816	0.022	0.773–0.860	74.6%	72.5%	<0.0001

AUC = area under curve; Std = standard; CI = confidence interval; MLR = monocyte to lymphocyte ratio; NLR = neutrophil to lymphocyte ratio; PLR = platelets to lymphocyte ratio; SII = systemic inflammatory index; SIRI = systemic inflammation response index; AISI = aggregate index of systemic inflammation; ALI = acute limb ischemia; ICU = intensive care unit.

**Table 4:** Univariate analysis of MLR, NLR, PLR, SII, SIRI, AISI, and the CT Severity Score and all patients’ adverse event occurrences during the study period.

	ALI	ICU	Mortality
**Low-MLR vs. high-MLR**	6/341 (1.76%) vs. 43/169 (25.44%) *p* < 0.0001 OR:19.05 CI: (7.91–45.86)	64/276 (23.19%) vs. 123/234 (52.56%) *p* < 0.0001 OR:3.67 CI: (2.51–5.36)	35/316 (11.08%) vs. 79/194 (40.72%) *p* < 0.0001 OR:5.51 CI: (3.50–8.67)
**Low-NLR vs. high-NLR**	12/411 (2.92%) vs. 37/99 (37.37%) *p* < 0.0001 OR:19.84 CI: (9.81–40.11)	39/252 (15.48%) vs. 148/258 (57.36%) *p* < 0.0001 OR:7.34 CI: (4.82–11.19)	15/297 (5.05%) vs. 99/213 (46.48%) *p* < 0.0001 OR:16.32 CI: (9.09–29.30)
**Low-PLR vs. high-PLR**	6/346 (1.73%) vs. 43/164 (26.22%) *p* < 0.0001 OR:20.13 CI: (8.36–48.50)	49/266 (18.42%) vs. 138/244 (56.56%) *p* < 0.0001 OR:5.76 CI: (3.86–8.60)	36/343 (10.50%) vs. 78/167 (46.71%) *p* < 0.0001 OR:7.47 CI: (4.71–11.83)
**Low-SII vs. high-SII**	12/411 (2.92%) vs. 37/99 (37.37%) *p* < 0.0001 OR:19.84 CI: (9.81–40.11)	63/338 (18.64%) vs. 124/172 (72.09%) *p* < 0.0001 OR:11.27 CI: (7.32–17.35)	20/328 (20%) vs. 94/182 (51.65%) *p* < 0.0001 OR:16.98 CI: (9.92–29.06)
**Low-SIRI vs. high-SIRI**	10/341 (2.93%) vs. 39/169 (23.08%) *p* < 0.0001 OR:9.93 CI: (4.81–20.47)	55/269 (20.45%) vs. 132/241 (54.77%) *p* < 0.0001 OR:4.71 CI: (3.19–6.95)	30/346 (8.67%) vs. 84/164 (51.22%) *p* < 0.0001 OR:11.06 CI: (6.81–17.94)
**Low-AISI vs. high-AISI**	10/375 (2.67%) vs. 39/135 (28.89%) *p* < 0.0001 OR:14.82 CI: (7.14–30.77)	60/282 (21.28%) vs. 127/228 (55.70%) *p* < 0.0001 OR:4.65 CI: (3.16–6.84)	29/344 (8.43%) vs. 85/166 (51.20%) *p* < 0.0001 OR:11.39 CI: (7.003–18.55)
**Low-CT Severity Score vs. high-CT Severity Score**	8/352 (2.27%) vs. 41/158 (25.95%) *p* < 0.0001 OR:15.06 CI: (6.86–33.07)	58/264 (21.97%) vs. 129/246 (52.44%) *p* < 0.0001 OR:3.91 CI: (2.66–5.74)	29/316 (9.18%) vs. 85/194 (43.81%) *p* < 0.0001 OR:7.71 CI: (4.79–12.41)

MLR = monocyte to lymphocyte ratio; NLR = neutrophil to lymphocyte ratio; PLR = platelets to lymphocyte ratio; SII = systemic inflammatory index; SIRI = systemic inflammation response index; AISI = aggregate index of systemic inflammation; ALI = acute limb ischemia; ICU = intensive care unit.

**Table 5:** Multivariate analysis of new adverse events occurred during the entire study period.

	ALI			ICU			Mortality		
	OR	95% CI	*p* Value	OR	95% CI	*p* Value	OR	95% CI	*p* Value
**Age > 70** **Male sex** **AH**	1.03	0.99–1.06	0.051	1.42	0.98–2.05	0.059	1.50	0.97–2.30	0.06
	0.63	0.41–0.97	0.003	0.59	0.41–0.85	0.006	0.62	0.41–0.95	0.02
	1.23	0.52–2.77	0.14	1.54	0.92–2.58	0.09	1.69	0.90–3.19	0.10
**AF**	2.85	1.56–5.20	<0.001	1.27	0.85–1.91	0.24	2.43	1.56–3.80	<0.001
**PAD**	2.31	1.26–4.23	0.006	1.12	0.78–1.61	0.52	3.61	2.32–5.61	<0.001
**H** **igh-MLR** **H** **igh-NLR** **H** **igh-PLR**	6.82	3.51–13.28	<0.001	3.67	2.51–5.36	<0.001	5.51	3.50–8.67	<0.001
	30.28	13.97–65.60	<0.001	7.34	4.82–11.19	<0.001	16.32	9.09–29.30	<0.001
	12.07	7.71–21.77	<0.001	5.76	3.86–8.60	<0.001	7.47	4.71–11.83	<0.001
**H** **igh-SII**	30.28	13.97–65.60	<0.001	11.27	7.32–17.35	<0.001	16.45	9.60–28.16	<0.001
**H** **igh-SIRI**	15.22	7.33–31.62	<0.001	4.71	3.19–6.96	<0.001	11.06	6.81–17.94	<0.001
**HI** **gh-AISI**	14.82	7.14–30.77	<0.001	4.65	3.16–6.85	<0.001	11.39	7.003–18.55	<0.001
**H** **igh CT Severity Score**	14.71	6.12–35.33	<0.001	4.98	3.33–7.44	<0.001	09.89	6.23–21.79	<0.001

AH = arterial hypertension; AF = atrial fibrillation; PAD = peripheral arterial disease; MLR = monocyte to lymphocyte ratio; NLR = neutrophil to lymphocyte ratio; PLR = platelets to lymphocyte ratio; SII = systemic inflammatory index; SIRI = Systemic Inflammation Response Index; AISI = Aggregate Index of Systemic Inflammation; ICU = intensive care unit.

**Table 6:** Characteristics of ALI patients and multivariate analysis of new adverse events.

	ALI Patients *n* = 49			Survivors *n* = 22			Non-Survivors *n* = 27		*p* Value
**Rutherford Classification**									
I, no. (%)	8 (16.33%)			7 (31.82%)			1 (3.70%)		0.02
IIA, no. (%)	13 (26.53%)			7 (31.82%)			6 (22.22%)		0.45
IIB, no. (%)	15 (30.61%)			6 (27.27%)			9 (33.33%)		0.64
III, no. (%)	13 (26.53%)			2 (9.09%)			11 (40.74)		0.02
**Side Involved**									
Unilateral, no. (%)	40 (81.63%)			19 (86.36%)			21 (77.78%)		0.44
Bilateral, no. (%)	9 (18.37%)			3 (13.64%)			6 (22.22%)		
**Arterial Segment Involved**									
Aorto-Iliac, no. (%)	6 (12.24%)			2 (9.09%)			4 (14.81%)		0.54
Femoral, no. (%)	13 (26.53%)			11 (50%)			2 (7.41%)		0.003
Popliteal, no. (%)	14 (28.57%)			6 (27.27%)			8 (29.63%)		0.85
Infrapopliteal, no. (%)	13 (26.53%)			2 (9.09%)			11 (40.74%)		0.02
Upper Limb, no. (%)	3 (6.12%)			1 (4.55%)			2 (7.41%)		0.68
**Outcome** **s**									
ICU, no. (%)	38 (77.55%)			14 (63.63%)			24 (88.89%)		0.04
**Multivariate analysis**									
	**ICU**						**Mortality**		
	**OR**		**95% CI**		***p* value**		**OR**	**95% CI**	***p* value**
**RC I**	0.20		0.04–1.02		0.054		0.08	0.009–0.73	0.02
**RC III**	1.83		0.34–9.88		0.48		4.72	1.17–18.52	0.04
**Femoral**	0.18		0.04–0.79		0.02		0.08	0.01–0.42	0.003
**Infrapopliteal**	4.61		0.52–40.27		0.16		6.87	1.32–35.57	0.02

ICU = intensive care unit; RC = Rutherford class.
